# Performance on sit-to-stand tests in relation to measures of functional fitness and sarcopenia diagnosis in community-dwelling older adults

**DOI:** 10.1186/s11556-020-00255-5

**Published:** 2021-01-08

**Authors:** Xianyang Sherman Yee, Yee Sien Ng, John Carson Allen, Aisyah Latib, Ee Ling Tay, Huda Mukhlis Abu Bakar, Chien Yee Jolene Ho, Wan Cheen Charissa Koh, Hwee Heem Theresa Kwek, Laura Tay

**Affiliations:** 1 Duke-NUS Medical School, 20 College Road, Singapore, 169856 Singapore; 2 Department of Rehabilitation Medicine, Singapore General Hospital and Sengkang General Hospital, Singapore, Singapore; 3 Geriatric Education and Research Institute, Singapore, Singapore; 4 Health Services Research and Evaluation, SingHealth, Singapore, Singapore; 5 Department of Physiotherapy, Sengkang General Hospital, Singapore, Singapore; 6 Sengkang General Hospital, Singapore, Singapore; 7 Deparment of Dietetics, Sengkang General Hospital, Singapore, Singapore; 8 Department of General Medicine (Geriatric Medicine), Sengkang General Hospital, Singapore, Singapore

**Keywords:** Sarcopenia, Sit-to-stand tests, Grip strength, Elderly, Functional fitness

## Abstract

**Background:**

The sit-to-stand (STS) test has been deployed as surrogate measures of strength or physical performance in sarcopenia diagnosis. This study examines the relationship of two common STS variants – Five Times Sit-to-Stand Test (5TSTS) and 30 s Chair Stand Test (30CST) – with grip strength, muscle mass and functional measures, and their impact on sarcopenia prevalence in community-dwelling older adults.

**Methods:**

This is a cross-sectional analysis of 887 community-dwelling adults aged ≥50 years. Participants completed a battery of physical fitness tests - 5TSTS, 30CST, grip strength, gait speed, Timed-Up-and-Go (TUG) for dynamic balance and six-minute walk test (6MWT) for cardiorespiratory endurance. Muscle mass was measured using multi-frequency segmental bioelectrical impedance analysis (BIA). We performed correlation analysis between STS performance and other fitness measures and muscle mass, followed by multiple linear regression for the independent determinants of STS performance.

**Results:**

Mean participant age was 67.3±7 years, with female predominance (72.9%). STS tests exhibited weak correlations with grip strength (30CST, *r* = 0.290; 5TSTS, *r* = − 0.242; both *p*< 0.01), and stronger correlations with gait speed (30CST, *r* = 0.517; 5TSTS, *r* = − 0.533; both p< 0.01), endurance (30CST, *r* = 0.558; 5TSTS, *r* = − 0.531; both *p* < 0.01) and dynamic balance (30CST, *r* = − 0.501; 5TSTS, *r* = 0.646; both p< 0.01). Muscle mass correlated with grip strength but not STS. In multiple regression analysis, all fitness measures were independently associated with 30CST performance. Performance in both STS tests remained independent of muscle mass. There was no significant difference in prevalence of possible sarcopenia diagnosis using grip strength or STS (30CST, 25.0%; 5TSTS, 22.1%; grip strength, 22.3%; *p* = 0.276). When both measures are used, prevalence is significantly higher (42.0%; *p* = 0.276). Prevalence of confirmed sarcopenia with inclusion of muscle mass was significantly lower using STS compared with grip strength (30CST, 4.6%; 5TSTS, 4.1% vs. grip strength, 7.1%; *p*< 0.05).

**Conclusion:**

In the sarcopenia construct, STS tests better represents muscle physical performance rather than muscle strength. Different subsets of population with possible sarcopenia are identified depending on the test used. The lack of association of STS performance with muscle mass results in a lower prevalence of confirmed sarcopenia compared with grip strength, but may better reflect changes in muscle quality.

## Introduction

Maintenance of functional fitness is necessary for avoiding mobility disability in an aging population. Muscle strength features as a key component of functional fitness, alongside aerobic endurance, flexibility and dynamic balance. Sarcopenia is characterized by age-related loss of skeletal muscle, and contributes to adverse health outcomes in older adults [1]. Muscle strength is regarded as the most reliable measure of muscle function and is the primary parameter of sarcopenia in the updated definition proposed by the European Working Group on Sarcopenia in Older People (EWGSOP2) [2]. While hand grip dynamometry has traditionally been used as a measure of muscle strength in the assessment of sarcopenia, lower body strength may better associate with functional activities and mobility tasks compared with grip strength, being necessary for Activities of Daily Living (ADLs) such as transfers, walking and stairs climbing [3]. From a broader perspective in Sarcopenia, optimal muscle quality as a primary physiological construct determines good muscle function. Muscle quality outcomes extend beyond strength and force production and includes intact metabolism, thermoregulation, signaling and myokine production [4]. Ideally, muscle assessments should also incorporate changes in muscle quality in aging.

Sit-to-Stand (STS) tests are used to evaluate lower body strength in the community [3, 5, 6], and are simple to set up, easily conducted by trained, non-medical people, and manageable in restricted spaces [7–10]. Test standardization protocols have been described [11], with normative values available both in Western and Asian populations [3, 5, 6, 12]. Two commonly used STS test protocols are the time taken to complete five repetitions (5TSTS) [13] and the maximum number of chair stands within 30 s (30CST) [5]. The 30CST allows assessment of a wide range in ability and facilitates longitudinal monitoring of rehabilitation, as the 5TSTS may be limited by a floor effect in individuals with moderate to severe mobility limitations preventing completion of five repetitions [5]. Both tests are well validated with good test-retest and inter-rater reliability, and cutoff values for predicting adverse outcomes are available [14, 15]. In addition, worse performance on STS tests are correlated with poorer muscle quality outcomes including ectopic fat infiltration in muscle (myosteatosis) from impaired muscle metabolism [4, 16].

However, there is conflicting literature behind the fundamental construct underlying the STS test. It is unclear if STS tests better reflect muscle strength or physical performance in the diagnosis of sarcopenia. While STS is accepted as a proxy for lower limb strength in the revised EWGSOP2 operational diagnosis of sarcopenia [2], it is considered a surrogate measure for physical performance alongside gait speed and the Short Physical Performance Battery (SPPB) in the recent Asian Working Group for Sarcopenia (AWGS 2019) guidelines [9, 17]. Nonetheless, both approaches are prevalent in literature. STS tests are still widely used as measures of strength [3, 6], and are well correlated with objective strength testing such as leg-press resistance [5] and power-rigs [18]. However, prior studies suggest that STS is not a mere proxy for muscle strength, but also reflects endurance, balance and mobility [19]. While the 5TSTS test has been demonstrated to be an appropriate surrogate for gait speed in sarcopenia diagnosis [9], no study has yet examined the utility of 30CST as a measure of strength or physical performance despite it being an easier to perform STS test-variant in the elderly.

We hypothesize that in the sarcopenia construct, the 5TSTS and 30CST have stronger correlations to physical performance measures compared to muscle strength measures. We further hypothesize that the prevalence of sarcopenia will be significantly different if the STS tests were used instead of grip strength tests in conjunction with muscle mass measurements on BIA. Finally, we also aim to determine predictors of STS performance with a comprehensive set of demographic, health, social, behavioral and physical assessment variables.

## Methodology

### Study population

The Individual Physical Proficiency Test for Seniors (IPPT-S) is an ongoing community-based programme designed to promote fitness and prevent or delay sarcopenia and frailty among older adults [20]. Eight hundred eighty-seven participants have since been recruited and included in this cross-sectional analysis. Ethics approval was obtained from Singhealth Institutional Review Board.

Eligible participants were aged > 50 years old, community dwelling and able to ambulate independently (with or without walking aids). Screening platforms were based at void decks of public housing blocks, senior activity centres and community clubs in the northeastern region of Singapore served by a regional healthcare facility. All participants completed a questionnaire-based multi-domain geriatric screen as well as physical fitness assessment administered by trained study team members. There were no exclusion criteria for participants as long as they were able to present themselves at the respective test centers and were well enough to undergo the fitness assessments.

### Sit-to-stand test

Both the 30CST and 5TSTS were used in this study, in accordance with established protocols [5, 21]. Participants were instructed to rise as quickly as possible from a seated position, with full body weight on the chair, to a standing posture, with their legs fully extended, while keeping their arms folded across their chest. The test administrator first demonstrated the execution of the test, and participants did not perform any physical test involving the lower limbs immediately prior to STS, to ensure performance would not be affected by fatigue. The 5TSTS measures the time taken, in seconds, to complete five repeated chair stands. The 30CST measures the maximal number of chair stands completed during 30 s of the test. Impaired performance on 5TSTS was defined using AWGS 2019 cut off > 12 s. Owing to the lack of establish cutoff on 30CST for sarcopenia diagnosis, we adopted the lowest quintile as indicative of impairment, in line with the approach of AWGS 2019 for weak handgrip strength [17].

### Grip strength

Grip strength was measured using a JAMAR Plus Hand Dynamometer (Sammons Preston, Bolingbrook, IL, USA), following the Southampton protocol [22] with 2 trials for each hand, and alternating sides during the test. The maximal reading from all trials was used for analysis. Weak grip strength was defined using AWGS 2019 cutoffs < 28 kg for men and < 18 kg for women [17].

### Timed-up-and-go

Dynamic balance was assessed using the Timed-Up-and-Go (TUG), in which participants had to rise from a chair, walk a distance of 3 m before turning and returning to a seated position [23]. Time taken, in seconds, was measured.

### Habitual gait speed

Habitual gait speed was measured based on time taken to walk 10 m at usual pace, with a 2-m acceleration and deceleration zone before and after the timed section of 10 m [24]. Slow gait speed was defined using AWGS 2019 reference value of < 1.0 m/s [17].

### Six minute walk test

Cardiorespiratory endurance was evaluated using the six-minute walk test (6MWT), in which participants had to walk as fast as they could along a 20-m path with encouragement throughout the test [25]. The total distance traversed in 6 min was recorded. Participants were allowed to rest at any time during the test if they reported significant fatigue or breathlessness.

### Short physical performance battery

Participants completed tests of side-by-side, semi-tandem and tandem balance, and were scored on the SPPB by applying established cut-offs based on tests of usual gait speed and time to complete five chair stands [13]. A score < 9 was indicative of poor physical performance.

### Skeletal muscle mass measurement

Appendicular skeletal muscle mass (ASM) was measured using multi-frequency segmental BIA (MC-780 M, TANITA, Tokyo, Japan). Appendicular skeletal mass index (SMI) was calculated by summation of fat-free lean mass from all 4 limbs, normalized by the square of height (ASM/height^2^). Participants with any metal implant were excluded from BIA. Measurements were taken before the commencement of physical tests and were taken, as per manufacturer’s manual, in a standing upright position with feet and hands in contact with base electrodes and grip electrodes respectively. No specific instructions pertaining to hydration were given to participants prior to the test however all participants were screened via a short questionnaire to ensure they were feeling well and their blood pressure were normal before the tests. We defined low muscle mass using AWGS 2019 cutoff values of < 7.0 kg/m^2^ and < 5.7 kg/m^2^ for males and females respectively [17, 26].

### Fat mass index

We included Fat Mass Index (FMI) measurements as a proxy measure of muscle quality. Good correlations between increases in overall body fat and myosteatosis have been reported [27]. Appendicular fat mass (AFM) was measured using multi-frequency segmental BIA (MC-780 M, TANITA, Tokyo, Japan) following BIA-validated methods described previously [28]. Appendicular fat mass index (FMI) was calculated by the summation of fat mass from all 4 limbs, normalized by the square of height (AFM/height^2^) [29]. FMI incorporates height and body composition therefore it is a better measure than body mass index in reflecting nutritional status and physical performance [28–30].

### Physical activity questionnaire

The type and amount of physical activity per week was recorded for all participants in a custom-designed questionnaire based on the Physical Activity Vital Sign Tool which in turn incorporates recommendations on physical activity from the American College of Sports Medicine (ACSM) and the American Heart Association (AHA) [31]. Activities were further categorized by intensity levels including walking or Tai chi (low intensity), bicycling or golf (moderate intensity) and swimming or jogging (high intensity). Participants who did other forms of physical activity that were not listed as examples were also collated and analyzed.

### Operational definitions of sarcopenia

Probable (EWGSOP2) or possible (AWGS 2019) sarcopenia is defined by low muscle strength (using handgrip or chair-stand test in EWGSOP2 and handgrip in AWGS 2019) or reduced physical performance (based on chair-stand test in AWGS 2019) [2, 17]. A definitive diagnosis of sarcopenia is confirmed by presence of low muscle mass in participants meeting criteria for possible/probable sarcopenia. Aligning with AWGS 2019 criteria for confirmed sarcopenia, we included gait speed and SPPB in addition to chair-stand test amongst the physical performance measures.

### Statistical analysis

Sample size was calculated using G*Power 3.1.9.4 [32, 33]. We projected a sample size of 779 seniors for an 80% power with a 5% type 1 error rate to detect a Pearson’s correlation coefficient as small as 0.10 between STS and grip strength performance.

Descriptive statistics used to summarize participant data were the mean, standard deviation, minimum and maximum values. Associations between skeletal mass index, strength and physical performance measures were examined using Pearson correlation coefficient. Two-way analysis of variance (ANOVA) was used to assess the main effects of gender and age on 30CST and 5TSTS performance. Chi square test of homogeneity was used to compare prevalence of sarcopenia diagnosed using different performance measures. To examine the physical construct underlying STS, we performed separate multiple linear regression for the outcomes of 30CST and 5TSTS, with strength (grip as a proxy), mobility (gait speed), endurance (6MWT) and balance (TUG) as independent variables, adjusted for age, gender, muscle mass, height and weight. All variables were entered simultaneously in multiple linear regression analysis incorporating the backward elimination variable selection approach, with statistical significance to enter/remove at *P* = 0.05/0.10. Statistical analyses were performed using IBM SPSS Statistics for Windows, version 25 (IBM Corp., Armonk, N.Y., USA).

## Results

### Demographics and physical performance measures (Tables 1, 2)

Eight hundred and eighty-seven (887) participants completed baseline assessment, with mean age of 67.3 ± 7.0 years, and female predominance (72.9%). Performance on 30CST, 5TSTS, TUG, habitual gait speed and 6MWT was similar in men and women (*p*> 0.05). Mean grip strength was significantly higher in men (31.7 ± 7.0 kg vs 21.6 ± 4.4 kg, *p*< 0.01). We stratified STS performance by gender and age groups (51–60, 61–70 and 71+ years old) (Table 2). While performance on both 30CST and 5TSTS was not influenced by gender, significant decremental performance was observed with increasing age (18.5 ± 0.4, 16.6 ± 0.2 and 13.7 ± 0.3 repetitions, *p*< 0.01; 8.2 ± 0.2, 9.7 ± 0.2 and 11.8 ± 0.3 s, *p*< 0.01) in the overall cohort. There was no significant interaction between age and gender on STS performance. The 20th percentile values for 30CST were 11 and 12 repetitions for males and females respectively. The 20th percentile values for 5TSTS were 12.4 and 12.5 s for males and females respectively.

**Table 1:** Summary of participant demographics and clinical characteristics

Variable	Overall (*n* = 887)	Men (*n* = 240)	Women (*n* = 647)	*P*^a^	*Min-Max*
Age, years	67.3 ± 7.0	68.7 ± 7.3	66.8 ± 6.8	< 0.05	51–100
Height, m	1.56 ± 0.79	1.65 ± 0.06	1.53 ± 0.06	< 0.05	1.31–1.83
Weight, kg	60.1 ± 12.0	65.9 ± 10.8	58.0 ± 11.7	< 0.05	29.6–112.3
BMI, kg/m^2^	24.5 ± 4.6	24.2 ± 3.8	24.6 ± 4.9	0.212	12.9–46.9
SMI (ASM/Height^2^)	6.8 ± 1.2	7.9 ± 1.2	6.5 ± 0.9	< 0.05	3.97–13.4
FMI (AFM/Height^2^)	3.3 ± 1.7	2.2 ± 1.0	3.7 ± 1.7	< 0.05	0–14.4
Grip Strength, kg	24.3 ± 6.9	31.7 ± 7.0	21.6 ± 4.4	< 0.05	6.2–48.9
30CST, Reps	16.0 ± 5.3	16.2 ± 6.0	16.0 ± 5.0	0.523	3–35
5TSTS, sec	10.1 ± 4.3	10.1 ± 4.3	10.2 ± 4.3	0.786	2.7–49.0
TUG, sec	10.1 ± 3.7	10.2 ± 0.2	10.0 ± 0.2	0.523	3.3–59.9
Habitual Gait Speed, m/s	1.3 ± 0.27	1.29 ± 0.29	1.28 ± 0.27	0.667	0.20–2.16
6MWT, m	468.6 ± 106.3	477.5 ± 117.7	465.5 ± 101.8	0.176	60.0–708.1
Physical Activity Hours/Week	11.8 ± 10.8	7.9 ± 7.6	13.2 ± 11.4	< 0.05	0–104.5

*BMI* body mass index, *SMI* appendicular skeletal mass index, *30CST* 30 s chair stands, *5TSTS* five times sit to stand, *TUG* timed-Up-and-Go, *6MWT* six-minute walk test

^a^ Independent sample T-test

**Table 2:** Sit-to-Stand tests performance across gender and age

	All (***N*** = 856)			Males (***N*** = 225)			Females (***N*** = 631)		
Age	N	30CST (reps)	5TSTS (secs)	N	30CST (reps)	5TSTS (secs)	N	30CST (reps)	5TSTS (secs)
**51–60**	145	18.5^a^ ± 0.4	8.2^a^ ± 0.2	26	19.0 ± 5.4	8.1± 3.6	119	18.4 ± 4.7	8.2 ± 0.2
**61–70**	455	16.6^b^ ± 0.2	9.8^b^ ± 0.2	116	17.4 ± 6.1	9.7 ± 4.3	339	16.3 ± 5.0	9.7 ± 0.2
**71+**	256	13.7^c^ ± 0.3	11.9^c^ ± 0.3	83	13.8 ± 5.1	11.2 ± 4.3	173	13.7 ± 4.3	12.2 ± 0.4
**Means**				240	16.2 ± 6.0	10.1 ± 4.3	647	16.0 ± 5.0	10.2 ± 4.3

*30CST* 30 s chair stands; *5TSTS* five times sit to stand

Note: Two-way ANOVA showed no significant interaction between age groups and gender for 30CST (*F* (2,846) =0.61, *P*=0.543) and 5TSTS (*F* (2,846) =1.07, *P* = 0.345*)*. The gender main effect was non-significant for 30CST (*F* (1,846) =1.92, *P* =0.166) and 5TSTS (F (1,846) = 1.16, *P* = 0.282). The age group main effect was significant for 30CST (*F* (2,846) = 41.16, *P*< 0.01) and 5TSTS (*F* (1,846) = 27.33, *P* < 0.01). Results of post hoc pairwise comparisons on age group means using Tukey HSD *P*< 0.05 are indicated by superscripted means in the respective columns corresponding to 30CST and 5TSTS. Means not differing significantly would have the same superscript—hence all age group means were significantly different

### Relationship of STS with functional fitness measures and muscle mass (Table 3)

Not unexpectedly, both STS variants were significantly correlated (*r* = − 0.677, *p*< 0.01). STS exhibited weak correlations with grip strength (30CST: *r* = 0.290 and 5TSTS: *r* = − 0.242, *p*< 0.01). While grip strength was moderately correlated with muscle mass (*r* = 0.442, *p* < 0.01), SMI was only very weakly and inversely correlated with 30CST while being unrelated to 5TSTS performance. Specifically examining the relationship between lower limb muscle mass and STS performance yielded similar findings, with lower limb muscle having no correlation with 5TSTS and a weak inverse relationship with 30CST (*r* = − 0.092, *p* < 0.05). Both STS tests were strongly correlated with habitual gait speed, dynamic balance on TUG and endurance on 6MWT, all of which were only moderately correlated with grip strength.

**Table 3:** Pearson correlations of Sit-to-Stand tests and grip strength with muscle mass and functional measures

	30CST	Grip Strength	Habitual Gait speed	6-min walk	TUG
30CST	1.000	0.290**	0.517**	0.558**	− 0.501**
5TSTS	− 0.677**	− 0.242**	− 0.533**	− 0.531**	0.646**
Grip Strength	0.290**	1.000	0.360**	0.367**	− 0.267**
	**Whole SMI**		**Upper Limb SMI**		**Lower Limb SMI**
30CST	−0.076*		− 0.131**		− 0.092*
5TSTS	0.053		0.087*		0.060
Grip Strength	0.442**		0.311**		0.290**

*SMI* appendicular skeletal mass index; *30CST* 30 s chair stands, *5TSTS* five times sit to stand, *TUG* timed-Up-and-Go

** *P* < 0.01; * *P* < 0.05

### Sarcopenia diagnosis (Table 4)

Prevalence of possible/ probable sarcopenia was approximately 1.5-fold higher when both grip strength and STS performance could be applied (weak grip or 5TSTS> 12 s or 30CST in lowest quintile), compared with the use of each measure in isolation (*p*< 0.01). In comparing diagnostic performance of grip strength versus STS tests, we observed no significant difference in prevalence of possible/ probable sarcopenia based on grip strength (22.3%), 30CST (25%) and 5TSTS (22.1%) (*p* = 0.276), although there was a decremental trend across 30CST, 5TSTS and grip strength amongst women (*p* = 0.055). Prevalence of confirmed sarcopenia was highest when applying criterion of weak grip strength or poor physical performance (STS, gait speed or SPPB) in the same algorithm (10.5%), declining across isolated measures of grip strength (7.1%), 30CST (4.6%) and 5TSTS (4.1%) (*p*< 0.01). Amongst men, the application of grip strength criterion yielded significantly higher prevalence of confirmed sarcopenia (9.1%) compared with STS measure (3.4–4.0%), being similar to the prevalence yielded using a combination of grip strength and physical performance measures (9.0%). There was no significant difference in prevalence of confirmed sarcopenia using either grip strength or STS in women.

**Table 4:** Prevalence of possible and confirmed sarcopenia

		Functional Measure				
**Possible/ Probable Sarcopenia**	**Grip Strength or STS (*n*=867)**	**30CST (*n*=852)**	**5TSTS (*n*=856)**	**Grip Strength (*n*=867)**	*p*^a^	*p*^b^
Overall	(364) 42.0%^b^	(213) 25.0%	(189) 22.1%	(193) 22.3%	< 0.01	0.276
Male	(95) 41.1%^b^	(51) 22.7%	(50) 22.2%	(64) 27.7%	< 0.01	0.314
Female	(269) 42.3%^b^	(162) 25.8%	(139) 22.0%	(129) 20.3%	< 0.01	0.055
**Confirmed Sarcopenia**	**SMI + Grip Strength or Physical Performance**^**c**^ **(*n*=721)**	**SMI + 30CST (*n*=710)**	**SMI + 5TSTS** **(*n*=714)**	**SMI + Grip Strength** **(*n*=719)**	*p*^a^	*p*^b^
Overall	(76) 10.5%^b^	(33) 4.6%	(29) 4.1%	(51) 7.1%	< 0.01	< 0.05
Male	(16) 9.0%^b^	(7) 4.0%	(6) 3.4%	(16) 9.1%	< 0.01	< 0.05
Female	(60) 11.0%^b^	(26) 4.9%	(23) 4.3%	(35) 6.4%	< 0.01	0.245

*30CST* 30 s chair stands, *5TSTS*  five time sit to stand, *SMI* skeletal mass index

^a^Chi-square test of homogeneity across all 4 groups

^b^Chi-square test of homogeneity comparing 30CST vs 5TSTS vs Grip strength

^c^Physical performance: Gait speed < 1.0 m/s or 5TSTS ≥ 12 s or Short Physical Performance Battery ≤ 9 [17]. Additional criteria of 30CST based on 20th percentile cut off was also used

### Physical fitness measures independently associated with STS (Table 5)

Measures of functional fitness independently associated with 30CST performance included grip strength, TUG, habitual gait speed and 6MWT. With the exception of gait speed, these measures were also associated with 5TSTS performance. Weight and fat mass index significantly impacted both 30CSTand 5TSTS performance. Examination of the standardized coefficients revealed TUG to exhibit the greatest influence on 5TSTS performance (*β* = 0.504, *p* < 0.001**)**, while 30CST performance was most strongly associated with 6MWT (*β* = 0.298, *p* < 0,001).

**Table 5:** Summary of multiple linear regression analysis to identify independent variables predictive of 30CST and 5TSTS

Variable	Univariate				Final Model^**a**^					
	5TSTS		30CST		5TSTS^**b**^			30CST^**c**^		
	Coefficients^**d**^ (95% CI)	***P***-value	Coefficients^**d**^ (95% CI)	***P***-value	Coefficients^**d**^ (95% CI)	β	***P***-value	Coefficients^**d**^ (95% CI)	β	***P***-value
Grip Strength, kg	−0.151 (−0.192, −0.110)	**< 0.001**	0.222 (0.173, 0.272)	**< 0.001**	−0.061 (− 0.115, 0.008)	**−0.098**	**0.025**	0.186 (0.114, 0.258)	**0.243**	**< 0.001**
TUG, s	0.760 (0.700, 0.821)	**< 0.001**	−0.724 (− 0.808, − 0.640)	**< 0.001**	0.593 (0.510, 0.676)	**0.504**	**< 0.001**	− 0.198 (− 0.322, − 0.074)	**−0.137**	**0.002**
Gait Speed, m/s	−8.394 (−9.292, −7.496)	**< 0.001**	9.992 (8.874, 11.110)	**< 0.001**				2.933 (1.164, 4.701)	**0.152**	**0.001**
6MWT, m	−0.021 (−0.024, − 0.019)	**< 0.001**	0.028 (0.025, 0.031)	**< 0.001**	−0.009 (− 0.012, − 0.005)	**−0.212**	**< 0.001**	0.015 (0.011, 0.019)	**0.298**	**< 0.001**
SMI (kg/m^2^)	0.189 (−0.075, 0.454)	0.160	−0.338 (− 0.663, − 0.013)	**0.042**						
FMI (kg/m^2^)	0.253 (0.063, 0.444)	0.009	−0.503 (− 0.727, − 0.278)	**< 0.001**	−1.155 (−1.494, − 0.816)	**−0.455**	**< 0.001**	0.547 (− 0.091, 1.184)	**0.176**	**0.093**
Weight, kg	0.034 (0.010, 0.058)	**0.006**	−0.066 (− 0.095, − 0.037)	**< 0.001**	0.169 (0.123, 0.215)	**0.470**	**< 0.001**	−0.124 (− 0.212, − 0.035)	**−0.281**	**0.006**
Height, cm	−0.021 (− 0.057, 0.016)	0.266	0.011 (− 0.033, 0.056)	0.616				−0.143 (− 0.225, − 0.061)	**−0.214**	**0.001**
Age, yrs	0.203 (0.164, 2.42)	**< 0.001**	−0.248 (− 0.296, − 0.200)	**< 0.001**	0.040 (0.002, 0.078)	**0.065**	**0.041**	− 0.085 (− 0.136, − 0.035)	**−0.113**	**0.001**
Gender, 1=F, 0=M	0.090 (−0.560, 0.740)	0.786	−0.278 (−1.077, 0.522)	0.495	2.594 (1.533, 3.654)	**0.268**	**< 0.001**	−1.825 (−3.256, −0.395)	**− 0.154**	**0.012**
Physical activity hours/week	−0.028 (− 0.055, − 0.002)	0.037	0.024 (− 0.009, 0.056)	0.156						

*SMI* appendicular skeletal mass index, *30CST* 30 s chair stands, *5TSTS* five times sit to stand, *TUG* timed-Up-and-Go, *6MWT* six-minute walk test, *F* females, *M* males, *FMI* fat mass index

^a^ Obtained using backward elimination multiple linear regression with *p* = 0.05/0.10 to enter/remove

^b^ 5STS model adjusted *R*^2^ = 0.480

^c^ 30STS model adjusted *R*^2^ = 0.426

^d^ Unstandardized Coefficient, B

## Discussion

Our findings suggest STS performance relates significantly to measures of grip strength, gait speed, endurance and balance. However, its weak correlation with grip strength suggests that the two measures are not equivalent strength test proxies of one another. We also build on existing sarcopenia literature, demonstrating muscle mass to be more strongly related to grip strength compared with a surrogate measure of lower body strength via STS.

STS tests were more strongly correlated with gait speed, endurance and balance compared with grip strength, an observation consistent with the reported association between STS tests and fastest gait speed [34]. STS is a functional maneuver which not only involves knee extensor strength [35, 36], but also trunk stability [37], sensation and balance [19]. Objective measures of lower extremity strength using isokinetic dynamometer have also been more strongly associated with customary gait speed compared with grip strength [38]. The generally weak correlation between grip strength and STS in our cohort is consistent with observed poor associations between objective measures of hand grip strength and knee extensor/flexor strength [38–40]. Caution is thus needed when using grip strength as a sole proxy measure for lower extremity strength or performance. Furthermore, a task such as STS obviously does not involve the muscles responsible for gripping, therefore the poor correlation between grip strength and STS performance should not come across as too surprising.

Muscle mass exhibited modest correlation with grip strength, and negligible association with lower limb strength as represented by STS tests. The dissociation between muscle mass and muscle strength with age has been previously described and attributed in part to intramuscular adiposity, shifting the attention towards muscle quality as a more clinically relevant determinant of physical performance in older adults [41, 42]. In addition, age-related decline in muscle mass and strength of the lower body is also more pronounced than that of the upper body, owing to reduced physical activities such as walking, running and stair-climbing that would expectedly have a greater impact on the lower body, and compensatory upper body movements such as use of arm muscles in routine activities such as rising from a chair [43]. In addition, greater deficits of muscle power relative to mass and strength have been reported in older individuals [43, 44]. The STS task has been used for measurement of muscle power, with demonstrated relationship to a spectrum of fitness measures [45]. The unexpected inverse correlation, albeit weakly, between STS performance and muscle mass may suggest reduced ability to fully recruit motor units for the force necessary to perform daily living tasks such as rising from a chair or stair climbing.

Although 30CST and 5TSTS were strongly correlated, their physical construct may not be identical. Both tests appear to be representative of muscle strength, being independently associated with grip strength. However, while 5TSTS was particularly dependent on dynamic balance, cardiorespiratory endurance was a stronger determinant of performance on 30CST. The standardized time protocol of 30 s requires participants to have sufficient physical endurance for continued repetitions as opposed to just the pre-determined five repetitions in 5TSTS. This is reflected in the wide variability of performance on 30CST, ranging from 3 to 35 repetitions in our cohort. However, this could also be due to the large sample size where wide variability of 5TSTS and other physical tests could also be seen. Our findings parallel the observed poorer correlation between 5TSTS and pulmonary function, compared with 30CST, in patients with chronic obstructive pulmonary disease [46]. Higher body weight in obese individuals is known to impair weight bearing activities and reduce exercise capacity [47], accounting for the negative impact of body weight on 30CST performance. Both STS tests were influenced by dynamic balance, albeit more so for 5TSTS than 30CST, plausibly because the latter is more fatiguing [48] such that the effect of balance, which declines with lower limb muscle fatigue [49] may become less significant. The association of 5TSTS and 30CST with various measures of functional fitness supports STS tests in representation of overall physical performance as opposed to muscle strength in isolation and in tandem, its influence by body functions (such as cardiovascular and neural systems) beyond skeletal muscle.

Generally, the FMI indices are lower in our cohort compared to other Asian populations, however the lower mean FMI values in females follow a similar trend [28]. Intriguingly, a higher FMI, not SMI, is significantly associated with poorer performance in the STS tests in both the univariate and multivariate models. This affirms previous literature that body fat or myosteatosis impairs muscle performance particularly in functional performance in STS tests and related lower limb physical assessments [16, 50]. This also indicates that the STS test may be a reasonable proxy in the evaluation of muscle quality. More importantly, the results suggest that the deleterious impact of sarcopenia may not be solely due to the loss of muscle mass but also from a reduction in muscle quality from the negative biomechanical and inflammatory impact of fat accumulation [29]. Future research to confirm these findings could evaluate other domains of muscle quality in the community with advanced technology. These include non-invasive, portable modalities such as quantitative musculoskeletal ultrasound to evaluate muscle structure [51] and near infra-red spectroscopy (NIRS) to characterize muscle oxidative potential as a proxy for mitochondrial capacity [52]. Increasing age is also correlated with poorer STS test performance as it may represent progressive diminishing muscle quality over time [53].

The introduction of possible/ probable sarcopenia to diagnostic algorithms by EWGSOP2 and AWGS 2019 seeks to raise awareness of sarcopenia for early lifestyle intervention and timely evaluation. The criterion of weakness by grip strength and poor performance on STS likely identifies different populations of older persons as having possible/ probable sarcopenia, given similar prevalence when applying either measure in isolation but significantly higher prevalence with availability of either measure in the diagnostic algorithm. This is likely because STS tests are more representative of overall physical performance than just strength itself. Overall, prevalence of confirmed sarcopenia with muscle mass measured via BIA was three to five times lower than respective diagnoses of possible/ probable sarcopenia, reinforcing observations that the loss of strength with age far exceeds the decline in muscle mass [54]. The lower prevalence of confirmed sarcopenia with both STS measures compared with grip strength in our overall cohort parallels the findings of a Korean cohort [55]. Further research is needed to identify physical differences between seniors differentially identified as sarcopenic based on performance measure applied. This also implies that single measures should not be used to identify sarcopenia as individual tests vary in sensitivity and specificity depending on how sarcopenia is defined [56]. Composite measures should be used as different tests are complimentary in their ability to detect different components of physical performance, strength and muscle mass. For example, the use of the SPPB to screen for sarcopenia had lower sensitivity if it was used alone without measures of muscle mass [57].

The similar prevalence of possible and confirmed sarcopenia identified by 30CST and 5TSTS suggests utility of the former in sarcopenia diagnostic algorithm, overcoming the floor effect of 5TSTS in older adults [5] and potentially facilitating better quantification if used as biofeedback. However, normative values and cutoffs will need to be specifically developed for its use in sarcopenia. Further, the choice of functional measure for sarcopenia diagnosis should be guided by its prognostic value for relevant clinical outcomes [58] and responsiveness to intervention [38, 59].

The strengths of this study include a well characterized cohort of community-dwelling older adults beyond the target sample size necessary for the primary aim, who completed a comprehensive battery of functional performance and objective muscle mass measures. However, several limitations are acknowledged. Our cohort of participants included only those who were ambulatory which may not be truly representative of all of the older adults living in the community. Older adults who may have health conditions which prevent them from travelling to the test centers are not represented in this study. The prevalence of sarcopenia within community dwelling older adults may therefore be under estimated. The exclusion of seniors who were residents of sheltered or nursing homes also limits generalizability of the findings. While upper limb strength was measured objectively using a dynamometer, we lacked comparative objective measure of lower limb strength.

## Conclusion

STS tests in older adults are influenced by strength, dynamic balance and cardiorespiratory endurance, and thus represent overall physical performance rather than mere muscle strength. Because of this, it also identifies a different subset of the population with possible sarcopenia that may not have been picked up by hand dynamometer testing (grip strength). The utility of a multi-test battery to assess sarcopenia will hence be more appropriate. Further research is needed to identify physical differences between seniors differentially identified as sarcopenic based on performance measure applied as subsequent diagnosis of confirmed sarcopenia is more prevalent with grip strength. Poor performance on STS may also be due to reduction in muscle quality rather than muscle mass. Further research to evaluate muscle quality in the community with advanced technology may provide a different perspective of the functional impact of sarcopenia beyond the traditionally described losses in muscle strength, performance or muscle mass.

## Data Availability

The datasets generated and/or analysed during the current study are not publicly available due to institutional restrictions but are available from the corresponding author on reasonable request.

## Abbreviations

STS: Sit-To-Stand
5TSTS: Five times Sit-to-Stand
30CST: 30 s Chair Stand Test
TUG: Timed-Up-and-Go
6MWT: Six-Minute Walk Test
EWGSOP2: European Working Group on Sarcopenia in Older People
AWGS: Asian Working Group for Sarcopenia
ADLs: Activities of Daily Living
BIA: bioelectrical impedance analysis
IPPT-S: Individual Physical Proficiency Test for Seniors
SPPB: Short Physical Performance Battery
ASM: Appendicular skeletal muscle mass
SMI: Appendicular skeletal mass index
FMI: Fat Mass Index
AFM: Appendicular Fat Mass
ACSM: American College of Sports Medicine
AHA: American Heart Association
